# Supplementary material for: Splice-Junction-Based Mapping of Alternative Isoforms in the Human Proteome
Source: Cell Rep. Author manuscript; Available in PMC 2020 Jan 15. (PMC6961840; doi:10.1016/j.celrep.2019.11.026)
Supplement: 2 [file NIHMS1546469-supplement-2.zip › DF1/PXD010154/Liver/Liver_63_PAF1_WGSGGPLLSHQREDGHRPNSHR.pdf]

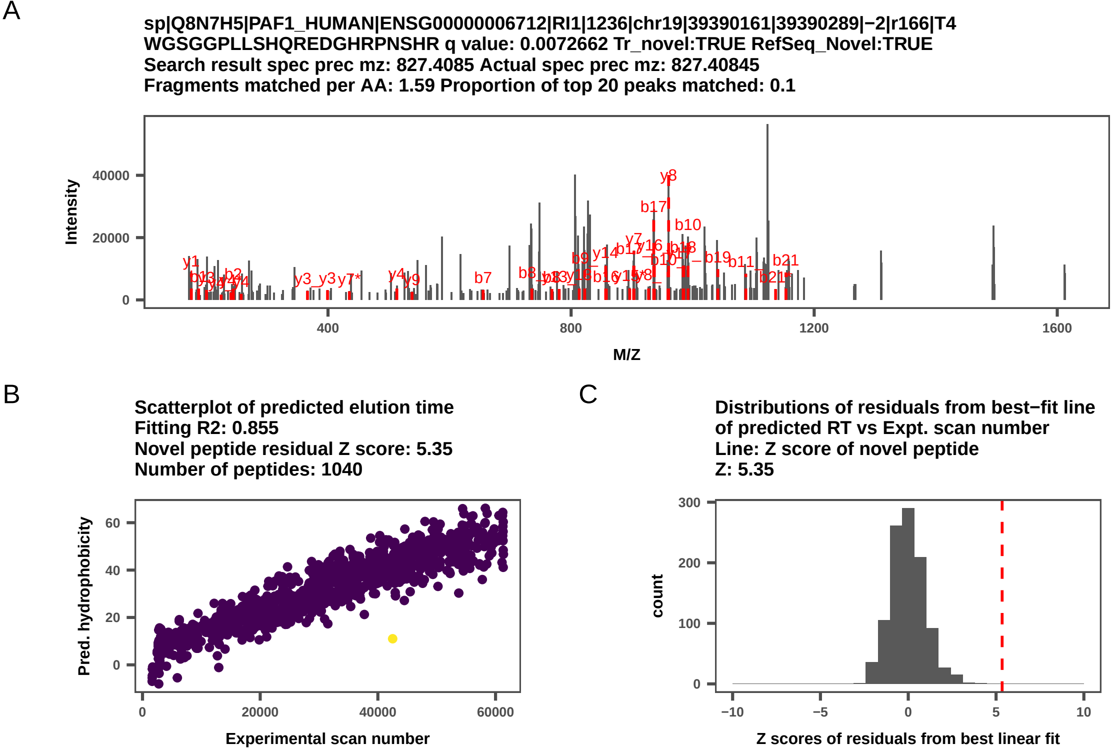
A
sp|Q8N7H5|PAF1_HUMAN|ENSG00000006712|RI1|1236|chr19|39390161|39390289|−2|r166|T4
WGSGGPLLSHQREDGHRPNSHR q value: 0.0072662 Tr_novel:TRUE RefSeq_Novel:TRUE
Search result spec prec mz: 827.4085 Actual spec prec mz: 827.40845
Fragments matched per AA: 1.59 Proportion of top 20 peaks matched: 0.1
Intensity
40000
20000
0
y8
b17
b10
y1
b7
b8
b19
b11
b21
400
800
1200
1600
M/Z
B
Scatterplot of predicted elution time
Fitting R2: 0.855
Novel peptide residual Z score: 5.35
Number of peptides: 1040
Pred. hydrophobicity
60
40
20
0
0
20000
40000
60000
Experimental scan number
C
Distributions of residuals from best−fit line
of predicted RT vs Expt. scan number
Line: Z score of novel peptide
Z: 5.35
count
300
200
100
0
−10
−5
0
5
10
Z scores of residuals from best linear fit
